# Faunal remains data from Paleolithic-early Iron Age archaeological sites in the Qinghai-Tibet Plateau in China

**DOI:** 10.1038/s41597-023-02858-w

**Published:** 2024-01-02

**Authors:** Kaidi Ren, Lele Ren

**Affiliations:** School of History and Culture, Lanzhou University, Lanzhou, 730000 China

**Keywords:** Archaeology, Agriculture

## Abstract

According to published archaeological sources, zooarchaeological data collection on the Qinghai-Tibet Plateau and its marginal and transitional areas is inadequate, and relevant datasets have not been published. For this reason, we collected and collated relevant information. Our database provides the geographical location, elevation, cultural type and faunal assemblage of each site on the Qinghai-Tibet Plateau and its periphery for which zooarchaeological data have been published from the Paleolithic to the Early Iron Age. The patterns of human faunal resource use, habitat patterns, and animal abundance and spatial distribution on the Qinghai-Tibet Plateau and its surrounding areas during the Prehistoric-Early Iron Age are represented in this dataset. The data provide a reference for further understanding prehistoric-early Iron Age human behavior, subsistence patterns and material and cultural exchanges between East and West on the Qinghai-Tibet Plateau and its environs.

## Background & Summary

The Qinghai-Tibet Plateau is the highest and largest plateau in China, known as the “roof of the world” and the “third pole of the earth”^1,2^, with an average altitude of approximately 4320 meters^3^. In China, it refers to the area bounded by the Kunlun, Altun, Qilian and Hengduan Mountains, including all of Tibet and Qinghai provinces and part of Gansu, Sichuan, Yunnan and Xinjiang provinces. The Qinghai-Tibet Plateau has a unique geographical environment and cultural tradition, and the process of human dispersal and settlement on the Qinghai-Tibet Plateau has always been a popular issue of widespread interest in academia^4–6^. The remains unearthed from archaeological sites can provide direct evidence for the study of this issue. Faunal remains can provide information for the study of human subsistence strategies, such as seasonal use of animal resources, as well as the origin of agriculture in the region.

The earliest dating results of paleolithic sites with buried cultural strata in the Qinghai-Tibet Plateau come from the Heimahe 1 and Jiangxigou 1 sites in the Qinghai Lake Basin^7^. The AMS ^14^C and Optically Stimulated Luminescence dating indicate that the two sites are approximately 15000–12000 BP. Stone tools such as stone cores, stone flakes, fine stone leaves, scrapers, fragments and detritus, and a small number of animal bones were unearthed at the sites of this period^7–9^. In the early and middle Holocene (11600-6000 BP), the archaeological cultures on the Qinghai-Tibet Plateau still present the appearance of paleolithic culture. Most sites of this period preserve a few stone artifacts, animal bones and individual fire pits, and a few sites have a thick cultural layer and rich unearthed remains^10,11^. During 6000-4000 BP, the Qinghai-Tibet Plateau entered the Neolithic period, and the number and spatial distribution of archaeological sites increased significantly. At the archaeological sites of this period, there were beaten and ground stone tools, a large number of potteries, bone objects, and faunal and plant remains. From 4000 to 2300 BP, agriculture and livestock farming economies developed and prospered on the Qinghai-Tibet Plateau^12^. The region entered the Bronze Age during this period. Many stone and bone production tools have been unearthed from large-scale sites.

However, there is a lack of published zooarchaeological data and a shortage of systematic collation of information in the Qinghai-Tibet Plateau region, and the existing data collection of archaeological sites in this region is insufficient in quantity and the available information is not comprehensive. Therefore, we collected archaeological site location information and faunal remains data from the Paleolithic to early Iron Age in the Qinghai-Tibet Plateau and its marginal and transitional areas with the Yunnan-Guizhou Plateau, Hengduan Mountain and Loess Plateau (25.650–40.212 N, 85.249–105.900 E). These data provide a basis for archaeologists, anthropologists, historians, and ecologists to study human migration and settlement, the exchange of civilizations, the use of resources, the domestication of animals and plants, and changes in the ecological environment. These data will play a positive role in enriching and exploring the evolution of civilization in the Qinghai-Tibet Plateau region, China and East Asia.

## Methods

**Fig. 1:**
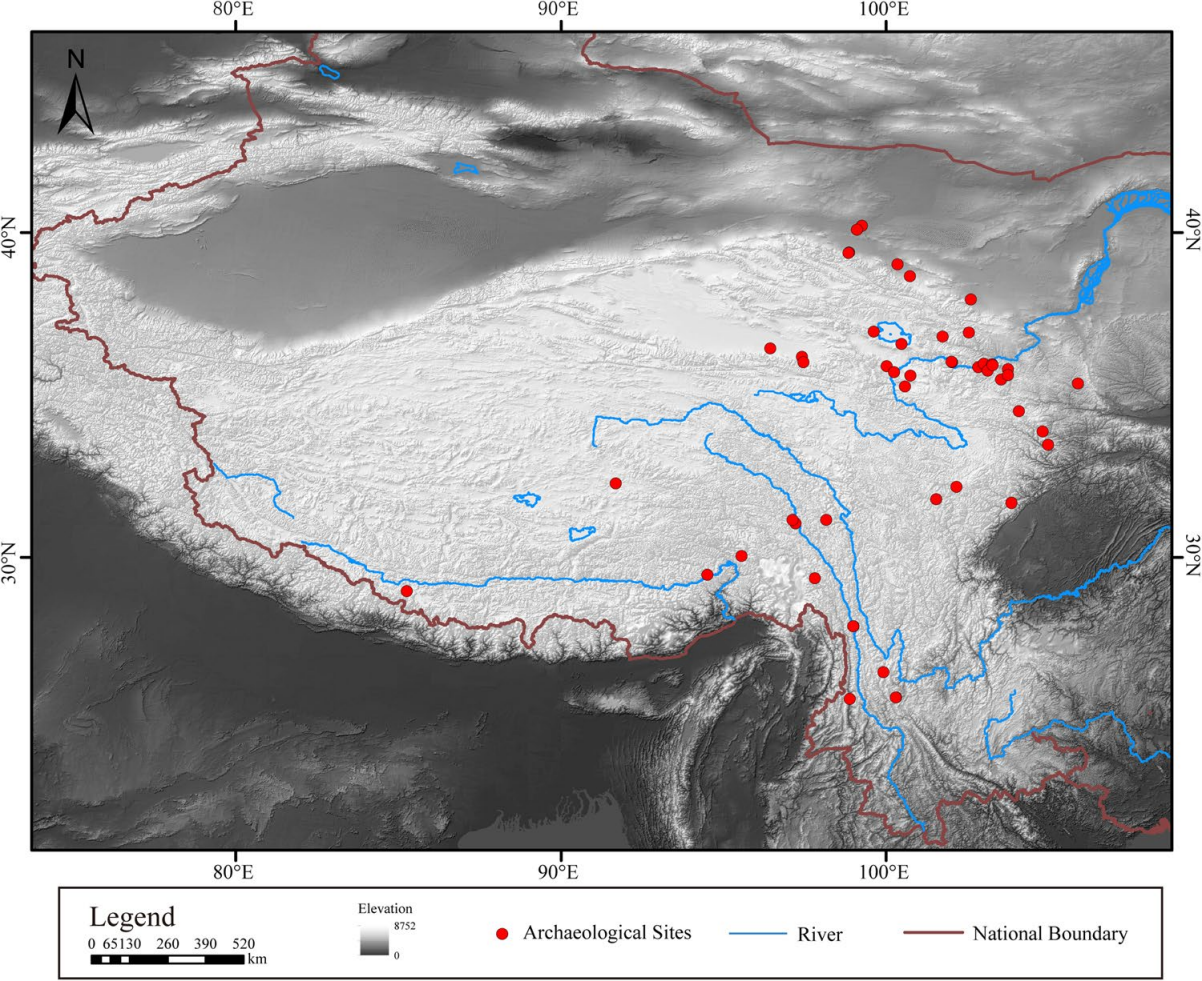
Archaeological site locations in China’s Qinghai-Tibet Plateau referred to in this study.

The site data used in this study are mainly based on the digitization of text data, which were obtained from archaeological excavations in the Qinghai-Tibet Plateau. Based on published faunal data and excavation reports^13–58^, data were collected from 47 sites. NISP (number of identified specimens) were collected from 40 sites, MNI (minimum number of individuals) from 31 sites and species identification data from 6 sites without specific data. The location of the site was calibrated with satellite imagery from the location description in the written records and then the archaeological excavation report was read carefully. Based on spatial location calibration, taking 30 m digital elevation in China as geographical background data, a location distribution map of archaeological sites was created with ArcGis10.8 (Fig. 1). The site date range is 15,000-1100 BP. There are two types of dates: radiocarbon dates and archaeological culture types.

Faunal identification in current zooarchaeological studies is mainly based on authoritative animal skeletal atlases^59–61^ and specimens in the collections of various laboratories. According to the identification report, we have divided the excavated faunal remains into three types: domestic animals, wild animals and domestic/wild animals. In addition to the bird ecological group “Wader”, 2 phyla, 7 classes, 17 orders and suborders, 40 families and subfamilies, 66 genera and 71 species were included, and they are presented in supplementary material Text S1.

**Fig. 2:**
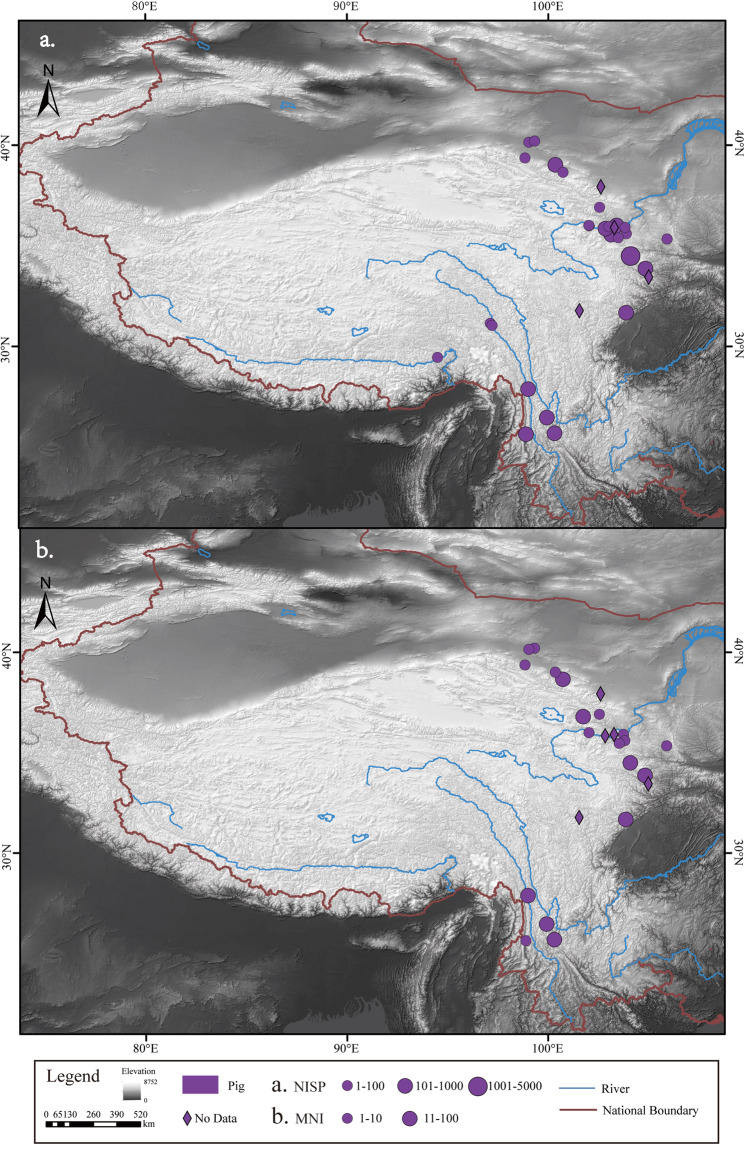
Spatial distribution of pigs unearthed at the archaeological sites. (**a**) NISP; (**b**) MNI.

**Fig. 3:**
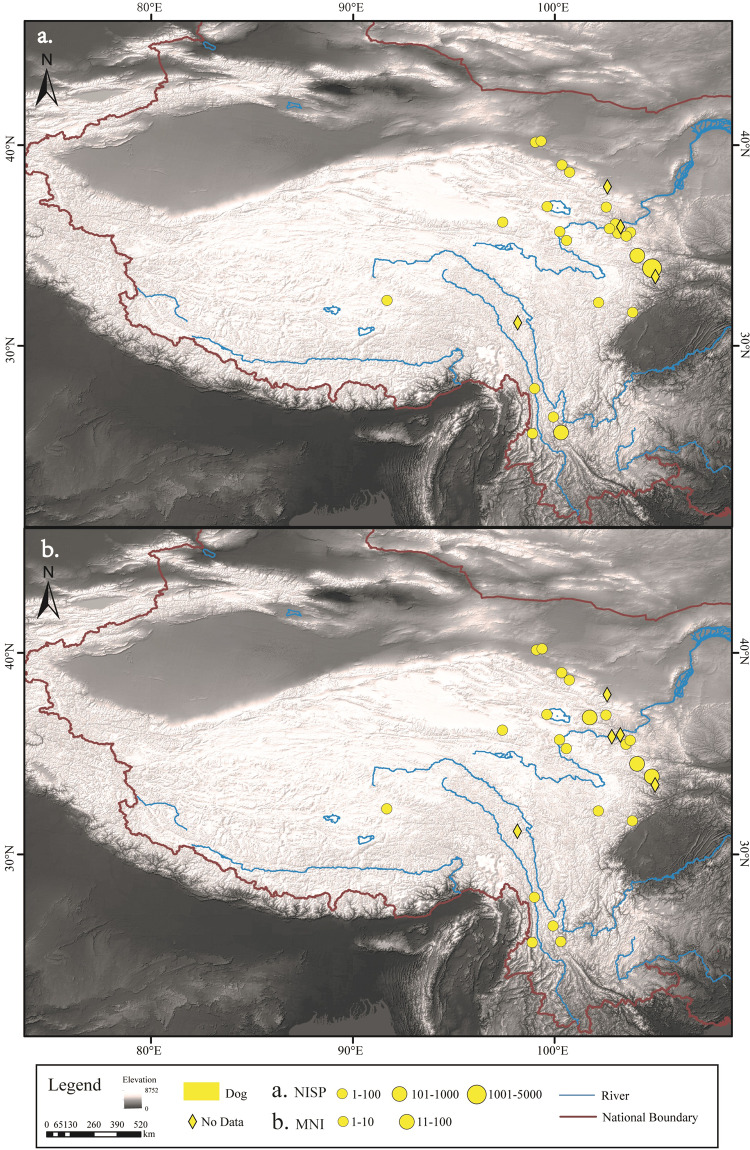
Spatial distribution of dogs unearthed at the archaeological sites. (**a**) NISP; (**b**) MNI.

**Fig. 4:**
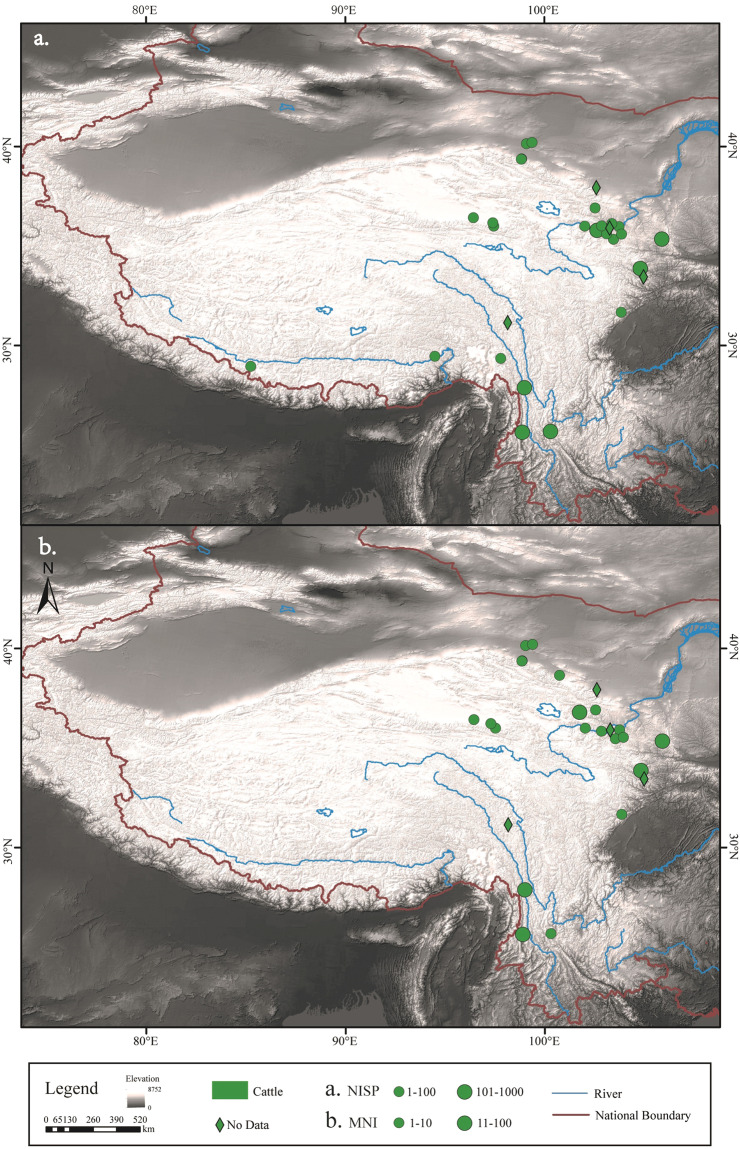
Spatial distribution of cattle unearthed at the archaeological sites. (**a**) NISP; (**b**) MNI.

**Fig. 5:**
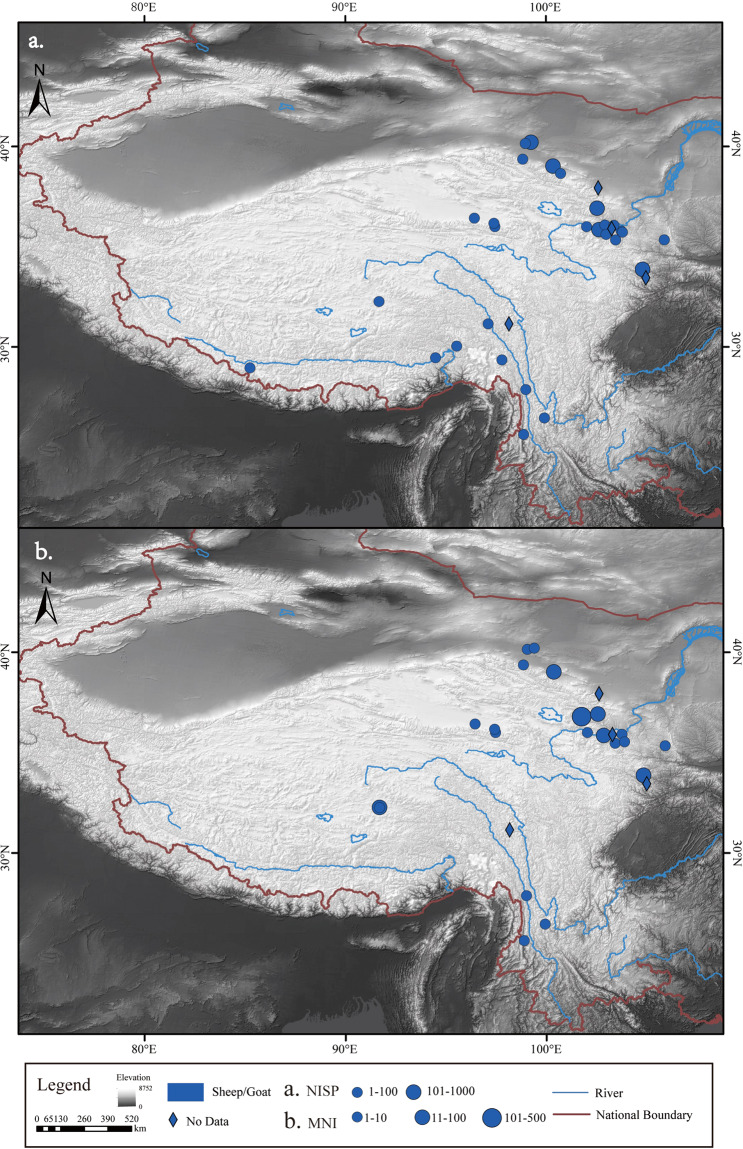
Spatial distribution of sheep/goats unearthed at the archaeological sites. (**a**) NISP; (**b**) MNI.

**Fig. 6:**
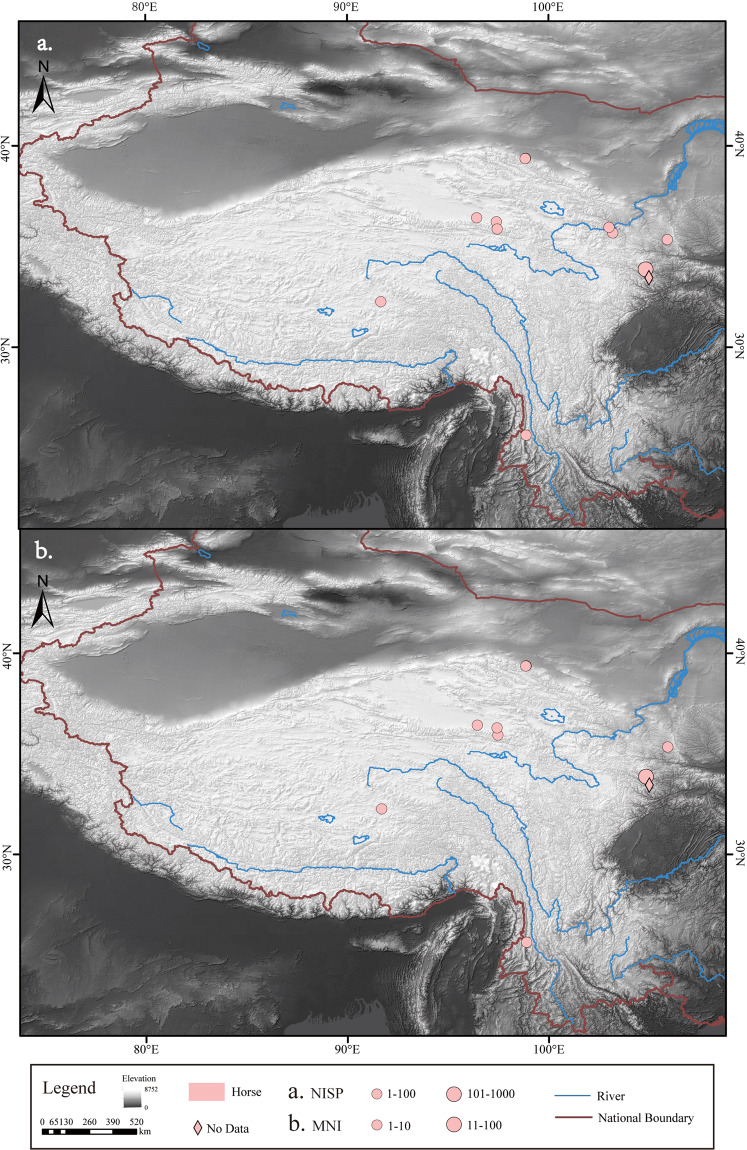
Spatial distribution of horses unearthed at the archaeological sites. (**a**) NISP; (**b**) MNI.

**Fig. 7:**
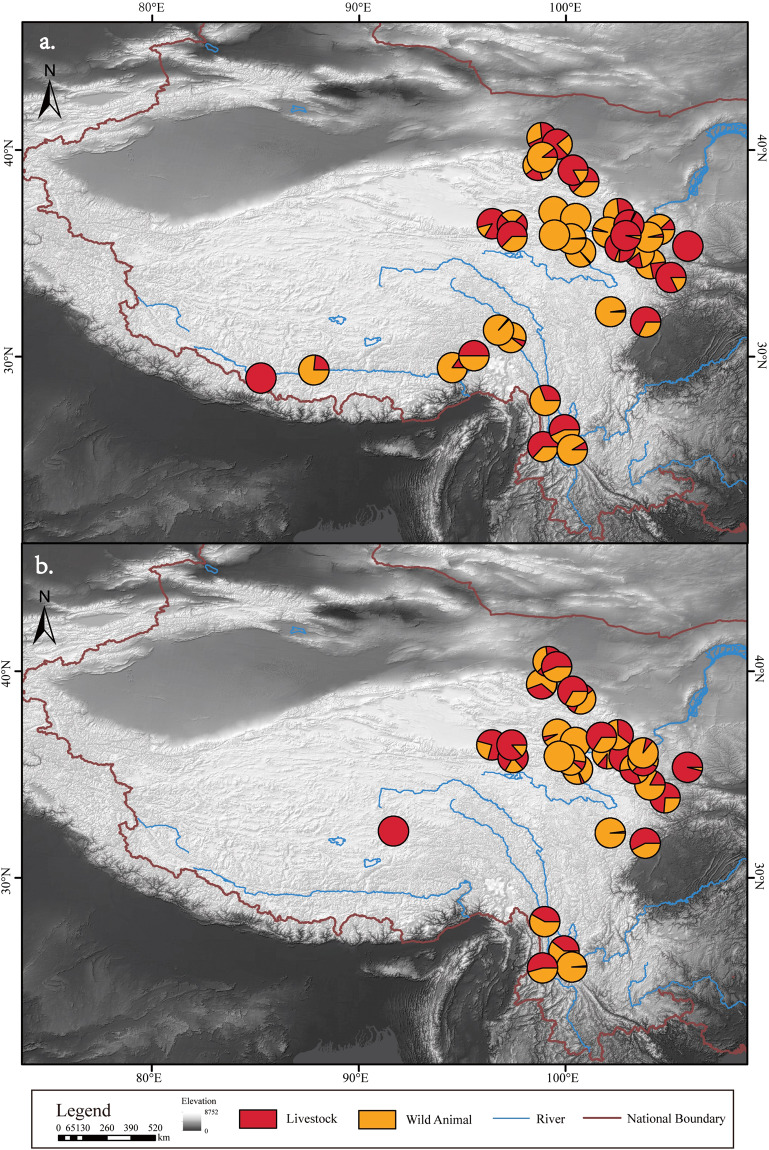
Spatial patterns of animal assemblages. (**a**) NISP. (**b**) MNI.

The number and distribution of NISP and MNI for each of the five common livestock species - pigs, dogs, cattle, sheep/goat and horses - were determined (Figs. 2–6). In addition, domestic animals and wild animals were distinguished and the NISP and MNI were mapped to scale (Fig. 7). These figures can be helpful for the study of the origin, domestication process and transmission route of domestic animals.

## Data Records

The raw data of this research have been released in Zenodo (https://zenodo.org/). 10.5281/zenodo.10025339^62^. The website is https://zenodo.org/records/10351442. The database is stored in the CSV and XLSX format in Zenodo (https://zenodo.org/). Eight basic pieces of information were collected for each site: (1) Location; (2) Name; (3) Longitude; (4) Latitude; (5) Altitude (m); (6) Culture type; (7) Age (a; BP); and (8) NISP and MNI. The “Location” is the administrative region where the site is located, and the “Name” represents the name of the site, based on the archaeological name of the site when it was discovered. The “Latitude” and “Longitude” are the longitude and latitude of the locations of cultural sites, which are recorded in decimal format. “Altitude (m)” refers to the altitude of the site or the region where the site is located. The altitudes and latitudes of some sites are recorded in archaeological excavation reports, while some sites were confirmed by satellite images, so they are approximate values. “Culture type” indicates the type of archaeological culture of a site. “Age (a, BP)” represents the age of a cultural site; the unit is a (year), BP is Before Present. There are no radiocarbon dates for the 9 sites at Yangqushierdang, Xiangranggou, Haxiu, Zhangjiazui, Zongzan, Qinweijia, Dalijiaping, Xishan, Jijiachuan and Huangniangniangtai. Radiocarbon dates were available for the remaining 38 sites. “NISP” means the number of identified specimens, and “MNI” means the minimum number of individuals, which are two quantitative indexes for determining specimen quantity statistics.

## Technical Validation

Information on the sites recorded in this study was obtained from written sources published by the State Administration of Cultural Heritage of China, the Qinghai Institute of Cultural Heritage and Archaeology, the Tibetan Institute of Cultural Heritage and Archaeology, the Gansu Institute of Cultural Heritage and Archaeology, the Yunnan Institute of Cultural Heritage and Archaeology, the Sichuan Institute of Cultural Heritage and Archaeology and the Chinese Archaeological Society. The zooarchaeological information was obtained from academic articles by archaeologists published in professional journals. For the extraction of geographical information, we used the highly recognizable geographical background data released by current official scientific research institutions. There is no problem with scientific and normative background data. However, the way in which the data were recorded varies between different eras, authors and institutions, and the lack of uniform standards for data recording inevitably leaves us open to the potential impact of these issues when collecting data information. Due to the lack of detailed records of in-site excavations, the cultural information recorded in the data is limited, and the spatial and temporal accuracy could be further improved. Most of the sites excavated earlier have only been preliminarily analyzed for faunal remains, which can bias judgments on numbers, species and domestication/wild. Due to these shortcomings, we are still collecting data and validating them as much as possible and constantly updating them and improving their quality. However, from the point of view of data application, these data are more used for macro analysis on large spatial scales than for discussion of micro site information, which requires highly precise location information. From this point of view, the spatial errors of the sites are understandable. The use of faunal remains data is also discussed more through the analysis of changes in faunal assemblages across time and space, and the impact of a few errors on the overall results is limited.

## Usage Notes

From what we have observed thus far, the application of these data is mainly in the following areas:

1. The subsistence patterns of prehistoric humans in the Qinghai-Tibet Plateau. Changes in faunal assemblages can provide evidence for the transition process from hunter-gatherers to a residential population in early humans. The variety and numbers of animals can also help to study the nature of the site.
2. The origin of domesticated animals. Based on the time and place of the appearance of domesticated animals, it is possible to determine the time of their introduction to China and the route of their spread, which can help in the study of the origin of domestic animals in China.
3. Paleoenvironmental reconstruction. Based on the species of wildlife and their habitats, the geography and climate of the Qinghai-Tibet Plateau region at the time can be recovered, thus inferring the environmental changes in the region.

### Supplementary information

Supplementary Information

## Data Availability

No code was used in the creation of these data. Titles should avoid the use of acronyms and abbreviations where possible. Colons and parentheses are not permitted.
