# Supplementary material for: Faunal remains data from Paleolithic-early Iron Age archaeological sites in the Qinghai-Tibet Plateau in China
Source: Sci Data. 2024 Jan 2;11:9. doi: 10.1038/s41597-023-02858-w (PMC10762139; doi:10.1038/s41597-023-02858-w)
Supplement: Supplementary file 1 — Supplementary Information [file 41597_2023_2858_MOESM1_ESM.docx]

Supplementary Materials for

**Faunal remains data of archaeological sites from the Paleolithic-early Iron Age in the Qinghai-Tibet Plateau in China**

Kaidi Ren^1^, Lele Ren^1, *^

1. School of History and Culture, Lanzhou University, Lanzhou 730000, China

* Corresponding author(s): Lele Ren (renlele@lzu.edu.cn)

Text S1. **Catalog of Life covered in this data set.** We have listed the species of animals involved in the data set and, for ease of inquiry, their corresponding parent taxa in this column.

Chordata

Aves

Galliformes

Phasianidae

*Phasianus*

*Phasianus colchicus*

*Alectoris*

*Alectoris chukar*

Falconiformes

Falconidae

*Falco*

Accipitridae

*Accipiter*

*Aegypius*

*Aegypius monachus*

*Buteo*

*Buteo hemilasius*

Anseriformes

Anatidae

*Anser*

*Anas*

*Anas platyrhynchos*

Passeriformes

Corvidae

*Corvus*

Mammalia

Artiodactyla

Suidae

*Sus*

*Sus scrofa domestica*

*Sus scrofa*

Ruminantia

Cervidae

*Cervus*

*Cervus elaphus*

*Cervus nippon*

*Cervus albirostris*

*Rusa unicolor*

*Axis*

*Axis porcinus*

*Capreolus*

*Capreolus capreolus*

*Capreolus pygargus*

*Muntiacus*

*Muntiacus reevesi*

*Muntiacus muntjak*

*Hydropotes*

*Hydropotes inermis*

Moschidae

*Moschus*

*Moschus moschiferus*

*Moschus berezovskii*

*Moschus chrysogaster*

Bovidae

Bovinae

*Bos*

*Bos taurus*

*Bos grunniens*

*Bos gaurus*

*Bubalus*

*Budorcas*

*Budorcas taxicolor*

Caprinae

*Ovis*

*Ovis aries*

*Ovis ammon*

*Capra*

*Capra hircus*

*Capra ibex*

*Pseudois*

*Pseudois nayaur*

*Naemorhedus*

*Naemorhedus goral*

*Naemorhedus caudatus*

*Rupicapra*

*Rupicapra rupicapra*

*Capricornis*

*Capricornis sumatraensis*

*Hemitragus*

*Hemitragus jemlahicus*

Antilopinae

*Procapra*

*Procapra gutturosa*

*Procapra picticaudata*

Perissodactyla

Equidae

*Equus*

*Equus ferus caballus*

*Equus asinus*

*Equus kiang*

Rhinocerotidae

*Rhinoceros*

Carnivora

Ursidae

*Ursus*

*Selenarctos thibetanus*

Ailuridae

*Ailurus*

*Ailurus fulgens*

Canidae

*Canis*

*Canis lupus*

*Canis lupus familiaris*

*Cuon*

*Cuon alpinus*

*Vulpes*

*Vulpes vulpes*

*Nyctereutes*

*Nyctereutes procyonoides*

Mustelidae

*Mustela*

Melinae

*Meles*

*Meles meles*

*Arctonyx*

*Arctonyx collaris*

Felidae

*Felis*

*Lynx*

*Lynx lynx*

*Prionailurus*

*Prionailurus bengalensis*

Viverridae

*Paguma*

*Paguma larvata*

Pantheriinae

*Panthera*

*Panthera tigris*

Rodentia

Muridae

*Rattus*

*Rattus rattus*

*Rattus norvegicus*

*Apodemus*

*Apodemus sylvaticus*

Gerbillinae

*Meriones*

Castoridae

*Castor*

*Castor fiber*

Hystricidae

*Hystrix*

*Hystrix brachyura*

Sciuridae

*Marmota*

*Marmota bobak*

*Marmoto himalayana*

Cricetidae

*Myospalax*

*Microtus*

*Volemys*

*Volemys millicens*

Rhizomyidae

*Rhizomys*

*Rhizomys sinensis*

Primates

Cercopithecidae

*Macaca*

*Macaca mulatta*

*Macaca thibetana*

*Rhinopithecus*

*Rhinopithecus roxellana*

Lagomorpha

Ochotonidae

*Ochotona*

*Ochotona princeps*

Leporidae

*Lepus*

*Lepus oiostolus*

*Lepus sinensis*

Actinopterygii

Cypriniformes

Cyprinidae

*Cyprinus*

*Cyprinus carpio*

*Gymnocypris*

*Gymnocypris przewalskii*

Amphibia

Anura

Ranidae

*Rana*

Reptilia

Testudinata

Emydidae

Trionychidae

*Amyda*

Mollusca

Gastropoda

Stylommatophora

Helicidae

*Helix*

Mesogastropoda

Viviparidae

*Bellamya*

Cypraeidae

*Monetaria*

*Monetaria moneta*

Lamellibranchia

Eulamellibranchia

Unionidae

*Lamprotula*

*Unio*

*Unio douglasiae*
